# The adaptation and evaluation of a culturally grounded lifestyle intervention to mitigate the risk of Alzheimer’s disease and related dementias in Native Hawaiians and Pacific Islanders: A study protocol

**DOI:** 10.1016/j.ssmmh.2025.100429

**Published:** 2025-03-17

**Authors:** Joseph Keaweʻaimoku Kaholokula, Dedra Buchwald, Richard MacLehose, Mele Look, Mapuana de Silva, J. Keʻalohilani Worthington Antonio, Kulani Desimone, Sheryl Yoshimura, Adrienne Dillard, Meghan Kenney, Chantelle Kealiʻihoʻomalu, Malia Purdy

**Affiliations:** a Department of Native Hawaiian Health, University of Hawaiʻi at Mānoa, USA; b University of Washington, USA; c Division of Biostatistics, University of Minnesota, Twin Cities, USA; d Hālau Mohala ‘Ilima, USA; e Kōkua Kalihi Valley Comprehensive Family Services, USA; f Kula no Nā Poʻe Hawaiʻi, USA; g Kapolei Community Development Corporation, USA; h Hui no Ke Ola Pono, USA

## 1. Introduction

### 1.1. Alzheimer’s disease in Native Hawaiians and Pacific Islanders (NHPI)

Native Hawaiians and Pacific Islanders (NHPI) in the United States (US) face a higher incidence and risk of Alzheimer’s disease and related dementias (ADRD) compared to other populations, with prevalence expected to rise in the coming years. The age-adjusted incidence of dementia among NHPI in the US is estimated at 20/1000 person-years, with a cumulative 25-year risk of 25 % at age 65 (Mayeda et al., 2016). A study of 6500 people with ADRD found the odds of early-onset ADRD among Native Americans (including Native Hawaiians) were 2.1 times higher than among Whites (Panegyres et al., 2014). In Hawai’i, the prevalence of ADRD is rising, with projections that 10 % of its population aged 65 and over will have an ADRD diagnosis by 2025 (State of Hawaii Department of Health, 2020).

The early onset of ADRD seen in NHPI is concerning due to its higher prevalence and its association with shorter survival and significant vascular risk factors compared to those with later onset in other populations (Siriwardhana et al., 2023). A neurodegenerative clinic-based study in Hawai’i reported that 70 % of their NHPI patients have a diagnosis of ADRD compared to 63 % of Asians, 53 % of Whites, and 65 % of all other patients (Smith et al., 2021). NHPI patients were considerably younger at diagnosis and presented with lower Mini-Mental State Exam scores than Asians and Whites. They also had more vascular risk factors for ADRD – hypertension (74 % vs. 55 % and 65 %, respectively), hyperlipidemia (70 % vs. 53 % and 61 %, respectively), and type 2 diabetes (28 % vs. 11 % and 22 %, respectively).

### 1.2. Pre-clinical manifestation and general risk of ADRD

In general, neurodegeneration due to ADRD begins biologically years before clinical dementia. Dementia is a clinically significant decline in cognitive function that interferes with daily activities and has various etiologies. ADRD progresses through stages: subjective cognitive impairment (SCI) or mild cognitive impairment (MCI), then clinical dementia (Albert et al., 2011). SCI is defined as a subjective report of worsening cognitive abilities (e.g., memory recall or orientation) without objective decline on standard cognitive tests. One study reported a 4.5 times higher risk of cognitive decline in people with SCI compared to those without subjective complaints (Reisberg et al., 2010). MCI is defined as either performance on cognitive function tests that is lower than expected relative to age or a clinically significant decline in performance that does not interfere with activities of daily life.

Among all populations, vascular, metabolic, and socioeconomic factors influence ADRD risk, while lifestyle interventions may help prevent or delay onset. Vascular and metabolic comorbidities are linked with ADRD, including hypertension (Debette et al., 2011), mid-life obesity (Loef and Walach, 2013), diabetes (Vagelatos and Eslick, 2013), and dyslipidemia (Meng et al., 2014). Low socioeconomic status, limited social and mental engagement, and a history of traumatic brain injury are also predictors of higher risk (Di Marco et al., 2014; Wang et al., 2012). Protective factors include lifestyle behaviors such as physical activity, healthy diet, cognitive activities, and social engagement (Lourida et al., 2013; Willis et al., 2012). ADRD disparities in minoritized populations are likely partly due to sociodemographic disparities (Chin et al., 2011). The NHPI community faces a high risk of ADRD due to the high prevalence of these vascular, metabolic, and socioeconomic factors in these communities.

### 1.3. NHPI and risk of ADRD

The NHPI population in the U.S. is growing and aging, with historical, demographic, and lifestyle changes contributing to increased ADRD risks. About 1.6 million people in the US identify as NHPI (Monte and Shin, 2022), reflecting a 29.1 % increase since 2010, and this number is expected to exceed 2.6 million by 2050. Although only 6 % of NHPI were aged 65 and older in 2020 (U.S. Census Bureau, 2020), this proportion is increasing as life expectancy in NHPI communities has risen from 74 to 83 years over the past decade (Monte and Shin, 2022). Cultural disruptions caused by foreign occupation and the militarization of the Pacific in the late 1800s led to a diaspora of NHPI to the US and other countries (Spickard et al., 2002). This resulted in a shift from traditional diets and behaviors to high-fat, high-sugar diets and sedentary lifestyles, which have increased their risk for chronic diseases (Wong and Kataoka-Yahiro, 2017).

NHPI face a higher burden of vascular and metabolic risk factors for ADRD, along with disparities in cardiovascular disease outcomes and treatment. Compared to non-Hispanic Whites, NHPI bear a higher burden of vascular and metabolic risk factors for ADRD, including hypertension, diabetes, obesity, dyslipidemia, CVD, and stroke (Mau et al., 2009). The CVD prevalence is nearly two times higher than non-Hispanic Whites (Waitzfelder et al., 2023). Compared to the all-races population, NHPI experience CVD and stroke an average of ten years earlier (Nakagawa et al., 2012) with mortality rates that are 68 % and 20 % higher, respectively (Johnson et al., 2004). NHPI are less likely to receive adequate treatment for cardiovascular conditions than Whites, and they are 30 % less likely to have controlled hypertension (Nakagawa et al., 2012).

### 1.4. Lifestyle intervention to prevent cognitive decline

The National Plan to Address Alzheimer’s Disease (Corriveau et al., 2017) recommends lifestyle interventions to treat, prevent, or postpone vascular contributions to ADRD in midlife to reduce the risk of dementia in later life. The most effective lifestyle interventions are those that target physical activity, healthy eating, and social and cognitive functioning. The Finnish Geriatric Intervention Study to Prevent Cognitive Impairment and Disability provided weekly physical activity, nutritional counseling, cognitive training, and management of metabolic and vascular risk factors over 24 months to 1260 participants aged 60–77 years at risk for ADRD (Kivipelto et al., 2013). This intervention improved or maintained executive functioning, processing speed, and neuropsychological test scores at 12 and 24 months compared to controls (Ngandu et al., 2015). A study involving 221 African Americans aged 66 and older with MCI indicated that a multi-domain lifestyle intervention effectively prevented cognitive decline over 24 months, with the greatest improvements observed at 12 months (Rovner et al., 2018). The intervention employed behavioral activation strategies to enhance physical activity and promote social and cognitive engagement. It included culturally sensitive activities, materials, and race-concordant community health workers to build trust. Although the behavioral activation strategies were less intensive than those in the Finnish study, they yielded similar results. Finally, a primary prevention trial involving 77 adults aged 50 and older revealed that goal-setting for healthy behavior change significantly improved memory and executive functioning over 12 months (Clare et al., 2015). To date, no intervention studies of ADRD or cognitive impairment have included NHPI (Toman et al., 2018).

### 1.5. Dance and cognitive function

Dance interventions can improve cognitive function, memory, and attention in older adults with early signs of cognitive impairment through physiological modifications. By integrating physical and mental exercises and social engagement, dance-based interventions can improve blood flow to the brain and the release of endorphins (Colcombe and Kramer, 2003), enhance neural connections and cognitive abilities (Kattenstroth et al., 2010), stimulate parts of the brain associated with social cognition and emotional regulation (Haslam et al., 2014), and reduce stress and improve mood through the release of endorphins and other mood-improving brain chemicals (Quiroga Murcia et al., 2010). A review of 14 dance interventions among older patients with MCI found significant enhancements in global cognition, rote memory, delayed and immediate recall, and attention (Liu et al., 2021). A study involving adults aged 60 and older compared an 8-month ballroom dancing program with a home walking program and optional group-based walking (Merom et al., 2016). The dance program resulted in greater improvements in visuospatial abilities and delayed recall at the 8-month follow-up. A review of five aerobic dance studies among older adults with MCI indicated that aerobic dance was linked to better global cognition, as well as enhanced immediate and delayed recall ability and executive function (Zhu et al., 2020)

### 1.6. Hawaiian cultural dance for health promotion

Hula is the hallmark of Hawaiian culture and the vehicle by which their native language, poetry, values, history, and connections to their natural and social world are preserved and passed on through the generations. Its popularity as a cultural dance form has extended beyond Native Hawaiians and is now practiced by people of diverse racial and ethnic backgrounds, ages, and genders. Through the coordinated use of arms and hands, legs and feet, and facial expressions – all following the beat and words of the music or chant – hula tells a story of heroic or beloved people, historical events, special places, or the genealogy of famous chiefs/chiefesses and the Hawaiian Islands. The songs or chants that accompany the hula are typically in poetry or narrative prose in which the dancer is the narrator. Hula involves longitudinal turns, head turns, shifting the center of gravity, and shifting balance, but it can be modified for people with limited physical capacity. Kumu hula (hula masters) are cultural experts who teach hula based on traditional cultural protocols. There are 193 *Hālau Hula* (Hula schools) in Hawai’i and 1100 schools that teach hula worldwide (Look et al., 2012).

Hula shows great potential for preserving cognitive function and preventing ADRD. Its energy expenditure averages a metabolic equivalent of 6 for moderate-intensity dancing and 8 for high-intensity dancing, respectively (Usagawa et al., 2014). A comparable activity at level 6 would be brisk walking, while level 8 is akin to running at 8 km/h. Hula also promotes interconnectedness with natural surroundings and fosters *‘ohana* (family) and *aloha* (compassion). A mind-body approach is emphasized through the values of *pono* (harmony) and *lōkahi* (unity) by spiritually connecting the dancer to places, people, and events. Hula also promotes *ho’okanaka* (cultural pride) and *kela* (excellence), which may improve mental health and encourage regular, long-term practice (Maskarinec et al., 2015).

A 6-month hula-based intervention significantly improved blood pressure and cardiovascular health in Native Hawaiian adults with hypertension. We previously tested a 6-month hula-based intervention, called *Ola Hou i ka Hula* (restoring health through hula), against a waitlist control for improving blood pressure in 263 Native Hawaiian adults (average age 56) with uncontrolled systolic hypertension (systolic ≥140 or ≥ 130 mmHg if diabetes) (Kaholokula et al., 2021). The first 3 months involved 2 60-min hula lessons per week, taught by a *Kumu Hula*, followed by 3 months of hula lessons and group sessions with a community peer educator that included heart health education and individualized plans to improve physical activity, diet, and stress management. Before randomization, all participants received an educational session. At the 6-month follow-up, the intervention participants showed greater reductions in systolic (−15.3 mmHg) and diastolic (−6.4. mmHg) blood pressure than controls (−11.8 and −2.6 mmHg, respectively), with 43 % of those in the intervention versus 21 % of controls reaching a hypertension stage of <130/80 mmHg. The reduction in 10-year CVD risk for the intervention group was twice that of the control group. All improvements for intervention participants were maintained at 12 -follow-up.

### 1.7. Study objectives

The ʻIKE Kupuna Project aims to adapt and evaluate a hula-based intervention designed to improve vascular health and cognitive function in NHPI elders who are at high risk of ADRD. As part of the Natives Engaged in Alzheimer’s Research (NEAR) network, funded by the National Institute on Aging to combat disparities associated with ADRD in American Indians, Alaska Natives, and NHPI, we proposed to implement the *‘IKE Kupuna* (Elder Wisdom) Project in partnership with five community-based organizations (CBO) in Hawai’i to adapt our existing hula intervention (*Ola Hou I ka Hula*) and test its effectiveness for improving vascular health and maintaining or improving cognitive function in NHPI elders at high risk of ADRD. Here, we present the protocols for adapting the hula-based program and the group-randomized trial to test its effects. The Specific Aims are as follows:

1. Adapt our existing hula-based intervention to target vascular risk factors for dementia, improve cognitive symptoms or function, and optimize salience, implementation, and adherence in NHPI adults with SCI or MCI. We expect the adapted intervention will incorporate culturally tailored education to promote a healthy diet, cognitive exercises, and an emphasis on community and social support.
2. Conduct a group-randomized trial (GRT) to test the effects of the adapted hula-based intervention on vascular risk factors for ADRD and cognitive complaints and function over 12 months in 144 NHPI elders. We expect the intervention to improve vascular risk factors, subjective cognitive complaints, and objective cognitive function compared to the wait-list control condition.

## 2. Material and method

### 2.1. Overview of study design

#### 2.1.1. Rationale

Culturally informed interventions, such as traditional dance, may help address the increasing risk of ADRD among aging NHPI individuals by promoting physical, cognitive, and social well-being. The NHPI population is growing and aging (State of Hawaiʻi, 2013), and the number of elders at risk for ADRD rapidly increases; however, NHPI individuals are profoundly underrepresented in ADRD research (Lim et al., 2020). Culturally informed interventions could be essential for improving health and eliminating the disparities these populations face. Traditional dance offers synergistic benefits through increased physical activity, emphasis on spatial awareness and neurocognitive stimulation, enhanced social connectedness, and a strengthened identification with one’s culture.

In partnership with five CBO in Hawai’i, we proposed an 8-month hula curriculum featuring cognitive exercises with Native Hawaiian language and songs, dietary education emphasizing eating patterns preferred by NHPI, and social and cultural engagement strategies. We include both Native Hawaiians and other Pacific Islander communities (e.g., Samoan, Tongan, Chuukese, and Marshallese) in this study, although the study is situated in the Hawaiian language and culture. This study is conducted on the traditional lands of the Native Hawaiian community and, as noted earlier, hula is popular among many other ethnocultural groups. An initial pilot study of the 3-month hula component of our intervention was previously tested with both Native Hawaiian and Chuukese participants and found acceptable and effective in improving hypertension management in both groups (Kaholokula et al., 201&). The current study aims to expand upon our previous research and is inclusive of all NHPI communities.

**Fig. 1.**
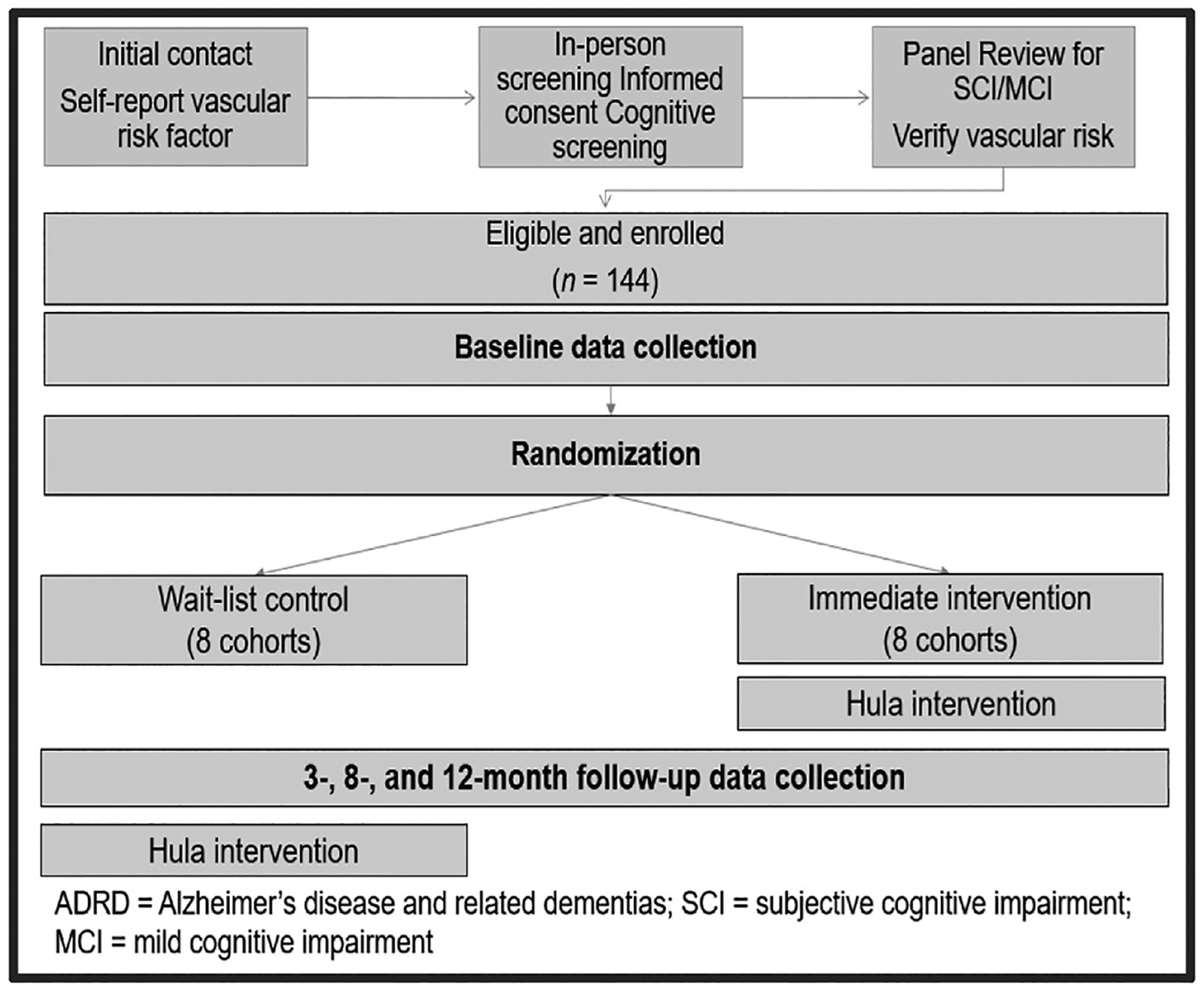
Group-randomized Hula trial.

We first use qualitative methods to refine the intervention. As Fig. 1 shows, we will then conduct a GRT with 144 NHPI aged 50–75 years who have prevalent SCI or MCI and at least 1 vascular risk factor for ADRD. Participants will be divided into 16 cohorts of 8–10 people each, then randomized by cohort to immediate intervention or a wait-list control condition. Primary outcomes are vascular risk factors for ADRD and subjective cognitive complaints. Secondary outcomes will be additional measures of cognitive performance, physical function, and mental health. Data collection will occur at baseline and at 3-, 8-, and 12-month follow-ups.

Our trial design is a partially nested GRT because randomization and delivery of the intervention occur at the group level for those in the intervention arm, but the control arm does not receive a group intervention (Murray et al., 2004). We chose the wait-list control group design and 12-month follow-up period to address our CBO partners’ requirement that a potentially beneficial intervention is not withheld from participants for longer than one year. As demonstrated by our previous study of hula for blood pressure control, 12 months is a reasonable time frame to observe improvement in vascular health, and other intervention studies have observed improved cognitive outcomes as early as 8 months.

#### 2.1.2. Conceptual grounding

The full title of our project is “Indigenous Knowledge and Experiences (‘IKE) Kupuna Project: Preventing Memory Loss in Older NHPI.” *‘Ike* is Hawaiian for knowing and experiencing; *Kupuna* means elder and ancestor. *‘Ike Kupuna* thus means ancestral knowledge, which speaks to our culturally grounded promotion of Hula to prevent cognitive decline and to our priority population of older NHPI at risk of ADRD. For NHPI, caring for elders and preserving their memory is vital to transmitting traditional cultural knowledge to ensure the health and well-being of future generations (Jackson et al., 2025). In Hawaiʻi, as elsewhere in the Pacific, elders serve as mentors to the youth, imparting a wealth of knowledge. This transmission is vital for the preservation of Hawaiian culture and Pacific culture in general.

We draw from the social cognitive theory (SCT) to inform our study, which emphasizes goal adoption for self-directed change, implementation strategies for productive actions, and maintenance strategies for sustainable behavioral changes by developing behavioral capability, self-control, and self-efficacy (Bandura, 2005). The Indigenist Stress-Coping Model (ISCM) foregrounds the critical need for lasting behavior change by leveraging Indigenous values that strengthen social identity and group support (Walters and Simoni, 2002).

ISCM aligns with the intent of ʻali Kūpuna, as both emphasize the role of cultural resilience and traditional practices in promoting well-being. By using hula as a preventative measure for vascular dementia, ʻ as Kūpuna draws on observational learning, self-efficacy, and cultural identity, which are core principles of SCT. The intervention strengthens cultural connection and social support, acting as a protective factor against stress-related health disparities while also reinforcing the intergenerational transmission of indigenous knowledge as a source of healing and empowerment for NHPI communities.

ISCM also explains how historical trauma, systematic oppression, and cultural displacement impact Indigenous health, while emphasizing cultural resilience and traditional practices as protective factors. It aligns with SCT by highlighting the dynamic interaction between personal, behavioral, and environmental influences, particularly through self-efficacy and observational learning within community settings. Because ʻIKE Kūpuna utilizes hula as a preventative measure for vascular dementia in NHPI populations, both SCT and ISCM help to explain its effectiveness. ISCM underscores how cultural identity and intergenerational knowledge transmission strengthen well-being, while SCT highlights how participation, role modeling, and social support encourage engagement and health behavior change.

**Fig. 2.**
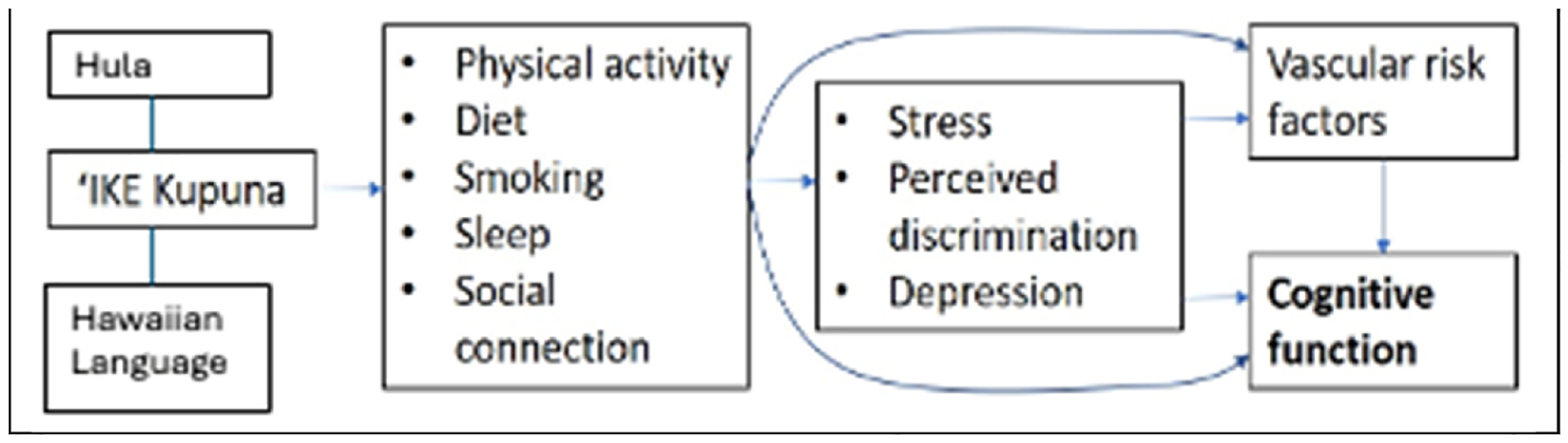
Conceptual model.

Fig. 2 depicts our conceptual model with the hypothesized relationships between study variables (Fig. 2). We expect that *‘IKE Kupuna* will lead to improvements in vascular ADRD risk factors and cognitive functioning through a causal path that is mediated by improvements in dance and other forms of physical activity; in diet, sleep, stress, and other psychosocial factors; and in cultural connection and social engagement.

#### 2.1.3. Community-academic investigative team

The investigative team is made up of research scientists, clinicians, and community leaders who collectively have the expertise and experience in NHPI culture and health to include hula, designing and testing culturally responsive and community-based lifestyle interventions to improve the vascular risk factors of ADRD in NHPI communities, and the measurement of cognitive functioning in diverse populations. The academic and clinical investigators include the Native Hawaiian translational behavioral scientists who served as the principal investigator for the trial that tested the effects of *Ola Hou i ka Hula* on hypertension management; an advanced hula practitioner with expertise in community engagement; the kumu hula expert who originally designed the hula components of *Ola Hou i ka Hula*; a neurologist who studies and treats patients with ADRD; and a methodologist experienced in conducting trials in Indigenous populations. The community investigators include leaders experienced in NHPI research who represent NHPI-serving where this project’s recruitment and implementation will occur, including community health centers, the Native Hawaiian Health Care System, and Hawaiian Homestead communities.

ʻIKE Kūpuna is a community-based participatory research (CBPR) project where the academic-based and community-based partners are part of the investigative team and share resources and decision-making. The development of the original hula intervention was led by cultural experts of hula from across Hawai’i to ensure its cultural integrity with co-leadership provided by several community leaders to ensure feasibility, which are detailed by Look et al. (2012, 2014). These cultural experts continue to serve as co-investigators for this project. Thus, our CBPR approach draws on a long-standing relationship with cultural experts and leaders of five community-based organizations, which include a Native Hawaiian Health Care System, Native Hawaiian Homestead communities, a community health center, and other health-related organizations. These community leaders and organizations are reflected in the co-authorship of this paper, which is consistent with our practice of co-production and co-dissemination of the research.

The cultural experts and community partners of ʻIKE Kūpuna actively participated as co-investigators in the design of the qualitative and quantitative aspects of this study. They will also actively participate in the implementation and evaluation of the intervention and all dissemination activities to include a report back to the community before any formal publication of results. The communities’ feedback on the qualitative findings and study design will shape the intervention and ensure it directly addresses the needs of their community and is culturally appropriate. To provide guidance and oversight, all academic and community-based investigators will serve on the project’s intervention Steering Committee. Simultaneously, capacity will be built in our community-based partners to ensure the intervention can be sustained beyond the life of this project by drawing our hula instructors for our intervention from the communities they serve and by training their community-based staff and community health workers to screen, enroll, and implement the intervention as well as to conduct all assessments.

#### 2.1.4. Intervention components

The primary intervention component consists of hula lessons led by a Kumu Hula (hula teacher) trained to implement the standardized intervention protocol, which will last for eight months. Months 1–3 include 2 60-min hula lessons per week (24 lessons total). Each lesson will follow a format that meets national recommendations for physical activity and is modifiable for people with limited mobility and fitness. In Months 4–8, the hula lessons are reduced to one lesson per week (20 additional lessons in total) to make room for the two other components.

The cognitive and social engagement aspect of the intervention capitalizes on the rich cultural elements of hula lessons, enhancing both mental stimulation and community connection. The hula lessons will promote NHPI values such as *lōkahi* (harmony with others and surroundings), *‘ohana* (family), and *aloha* (compassion). In learning the complex dance choreography, participants will learn the cultural meaning of the movements and memorize accompanying chants and songs in the Hawaiian language. Promotion of Hawaiian language will be fostered through a project-based learning approach in which Hawaiian words and phrases are used in class to direct dancers, and written translation of songs and chants and worksheets to promote hula-related vocabulary. Language learning has been shown to improve memory and cognitive adaptability (Kroll and Bialystok, 2013), and bilingualism has been shown to improve attention, task-switching, and cognitive flexibility (Bialystok et al., 2012). Listening to music engages sensory-motor processing, improves cognitive components, assists with memory, and has ties to emotional components (Zaatar et al., 2023). Many NHPI are not fluent in Hawaiian because of past acculturation strategies that banned its use in virtually all public settings and discouraged traditional language acquisition. In Months 4–8, other cultural lessons will be provided, such as traditional weaving, ukulele lessons, and Hawaiian language lessons and games, twice a month for 30 min in duration (10 lessons in total).

For the lifestyle behaviors component, intervention participants will receive culturally tailored lessons based on the Diabetes Prevention Program’s lifestyle intervention focusing on healthy diet, physical activity, and stress management during months 4–8 (Kaholokula et al., 2014). These lessons will be delivered by a trained community peer educator to each hula cohort. The dietary curriculum focuses on increasing fruit and vegetable consumption, reducing sodium and fat intake, portion control, understanding nutrition labels, developing healthy food shopping strategies, and managing eating at social occasions. The emphasis is on foods traditionally favored by NHPI, which includes an array of nutritious foods, including fruits, such as bananas and passion fruit, leafy and starchy vegetables, such as taro and ulu (breadfruit), and seafood. These traditional food items are locally sourced and available. The lessons also encourage engaging family and friends and other resources to support healthy eating and exercise habits. Participants set individual goals and learn how hula can help them maintain their lifestyle changes in the long term. These lessons will be delivered every other week, alternating with the cultural lessons, in months 4–8 and last 30 min in duration.

### 2.2. Protocols for intervention adaptation (specific aim 1)

#### 2.2.1. Qualitative investigation with NHPI participants and experts

Older adult participants will be recruited from our past trials of hula for hypertension, along with hula instructors and providers who treat NHPI with cognitive impairment. We will convene three focus groups with 15 NHPI aged 50 and older who participated in one of our past hula intervention projects. There are no other inclusion criteria. The only exclusion criterion is the presence of a significant cognitive problem (i. e., diagnosed dementia) that may prevent a participant from fully understanding the consenting process and the questions. Eligible participants will respond to semi-structured questions about their experiences with hula for health promotion (benefits, barriers), their thoughts about its use for physical, social, and cognitive engagement, and their recommendations for enhancing the social and cognitive aspects of the intervention for older NHPI. Each focus group will last about 90 min and be co-facilitated by a member of our investigative team and a peer educator from the CBO hosting the group. Each focus group participant will be provided a gift card valued at $25 as remuneration. It is not a requirement for these older adult participants to have SCI or MCI because the primary purpose of conducting these focus groups is to yield information to ensure the physical aspects of hula can accommodate the range of physical capacities for older adults.

Semi-structured key informant interviews will be conducted with four geriatricians, nurses, and/or other providers of local healthcare systems who care for NHPI with dementia. Also, four hula experts from the participating CBO who have experience with our hula intervention will be interviewed to elicit advice regarding the use of hula for improving cognitive function in older NHPI. ADRD advisors and hula experts will be provided information on the intervention curriculum and its efficacy in treating hypertension before interviews. They will be asked their opinions on the intervention components; their effects on exercise, social, and cognitive engagement; their importance in preventing ADRD; and any recommendations for enhancing the intervention for adults with memory problems. The hula experts will be asked how to maintain the cultural integrity of the intervention while enhancing retention and its social and cognitive components through hula-specific strategies. Each participant interviewed will be provided a gift card valued at $50 as remuneration.

#### 2.2.2. Qualitative data analysis

Framework analysis will be used to analyze findings (Klingberg et al., 2024). It is a structured approach to analyzing qualitative data, often used in health research, and useful when researchers have specific questions to generate practical insights from interviews and focus groups. Focus groups and interviews will be audio recorded for transcription by trained transcribers and analyzed along with field notes. A transcript-based, investigator-derived framework method to analyze focus group discussions and interviews will extract common themes, ideas, and recommendations and organize them into categories developed *a priori* (Gale et al., 2013). At least two research team members with hula expertise, Hawaiian cultural training, and/or qualitative research experience will separately review transcripts to identify items for the general domains of the framework and then repeat data, noting those that occurred with high frequency. They will meet to collaboratively review the information extracted and establish an agreement on key information provided and their appropriate domain in the framework. An investigator with Hawaiian cultural and hula expertise will review the transcripts to determine if additional information and/or domains could be identified or clarified. These findings will be used to refine the hula intervention. The final protocol will be reviewed and approved by the Steering Committee.

### 2.3. Protocols for group-randomized trial (specific aim 2)

#### 2.3.1. Target population and recruitment

After intervention adaptation, we will recruit NHPI aged 50 and older with SCI or MCI and at least 1 modifiable vascular or metabolic risk factor. Inclusion criteria: 1) self-reported NHPI ancestry, 2) age 50–75 years, 3) SCI or MCI, 4) diagnosis of hypertension, diabetes, dyslipidemia, or obesity measured at enrollment, 5) willing and able to engage in the moderate physical activity necessary for hula, and 6) physician’s approval to participate in moderate physical activity. These specific inclusion criteria are intentional as this is the optimal age range for preventing future dementia in people with cognitive impairment. Those above 75 years old are not likely to benefit from this study, given their advanced age. Exclusion criteria: 1) actively practicing in hula at least weekly, 2) normal cognition and no subjective cognitive complaints, 3) diagnosed ADRD (mild to severe), or 4) currently diagnosed major depressive disorder at moderate or greater stage.

The recruitment sites will adhere to the same recruitment strategy, and each site will recruit participants for up to two hula cohorts (16 total with concurrent intervention and control cohorts at each site). CBO staff will use their records to identify potentially eligible NHPI based on client registries and/or lists of past recipients of services. They will be contacted by mail or by phone to inform them about the study and asked to contact the CBO research coordinator if they are interested in participating. CBO staff will also set up information booths at events (e.g., health screenings and cultural gatherings). People who approach the booth will be given a card similar to that mailed to potential participants in step 1, with the option of completing the brief screening. Individuals interested in participating will complete a brief screening form to determine preliminary eligibility, which will include questions about a family history of ADRD, any previous clinical MCI or ADRD diagnosis, and having noticed or been told by others that their memory is getting worse or that they are getting “more scattered.” Endorsing at least one of these items will meet the requirements for further screening to determine SCI or MCI eligibility.

#### 2.3.2. Informed consent and screening

Potentially eligible participants will be scheduled for an in-person enrollment visit at the CBO, at their home, or at another location that offers sufficient privacy to conduct the interview. At this visit, we will verify the presence of at least 1 qualifying vascular risk factor. For high blood pressure, diabetes, and dyslipidemia, participants will be asked to either produce a pill bottle or other proof of a current prescription or to consent to have study staff verify the diagnosis through their healthcare provider. For obesity, we will measure height and weight to verify a body mass index ≥30 kg/m^2^. The Research Coordinator will then review the study’s goals, procedures, and requirements for participation. Written informed consent will include a box that participants can initial to indicate their understanding that final enrollment depends on confirmation of criteria for a vascular risk factor and SCI or MCI.

To date, there are no published cognitive assessment tools validated for NHPI. Since it is not yet possible to use biomarkers as the clinical gold standard to assess the presence of ADRD in individuals, a combination of three assessment tools was used to assess an individual’s cognitive performance. To screen for SCI/MCI, staff will first administer the Cognitive Change Index (CCI), a subjective measure of cognitive impairment (Rattanabannakit et al., 2016). It contains 20 questions scored on a 0–4 Likert scale (range 0–80), with higher scores representing more subjective memory complaints. Scores ≥6 will indicate prevalent SCI. Second, staff will administer the Quick Dementia Rating System (QDRS), a validated staging assessment for ADRD of participants’ self-reported ratings of cognitive function (Galvin, 2015). Ratings are based on the extent and severity of change from prior abilities in 10 domains such as Memory and Recall, Decision Making/Problem Solving, and Activities Outside the Home. Scores range from 0 to 30 and will be categorized using established thresholds (0–1.5 = normal; 2.0–5.5 = MCI; 6.0–12.5 = mild ADRD; 13.0–20.5 = moderate ADRD; ≥21 = severe ADRD) (Galvin, 2015). Third, the NSCT will be administered, a brief 90-s executive task that incorporates attention, planning, and set-switching. The scoring is from 1 to 70, using the number of correct answers. The test uses a cut-off score of 36 or greater to indicate normal cognitive function. Fourth, the 2-item PHQ (Irwin et al., 1999) will be used to screen for depression, which can contribute to false positive results in screening protocols for cognitive impairment.

To determine each participant’s cognitive impairment eligibility, the scoring scheme based on the CCI, QDRS, and NSCT scores summarized in Table 1 will be used. It shows how a research-only diagnosis of normal, SCI, MCI, or ADRD will be used to assess inclusion criteria #3 and exclusion criteria #2 and #3. The decision to use these three cognitive function measures and the scoring scheme in Table 1 was based on recommendations from our ADRD expert, a nationally-recognized neurologist. Because there have not been any validation studies of existing cognitive functioning measures validated for use among NHPI, we decided to use multiple measures of cognitive functioning in research to ensure a more comprehensive (i.e., each measure assesses different aspects of cognitive functioning) and reliable assessment of SCI and MCI. The PHQ2 will assess exclusion criteria #4. Once eligibility is confirmed, participants will be given a standardized form to provide to their physicians requesting their approval to engage in moderate physical activity confirmation of a diagnosis of hypertension, diabetes, and/or hyperlipidemia to address inclusion criteria #4 and #6.

#### 2.3.3. Randomization, data collection, and safety monitoring

After obtaining informed consent and confirming eligibility, participants will be scheduled for baseline data collection. Eligibility screening cognitive data will be used as baseline values for those measures. Aside from these cognitive measures, other clinical and behavioral measures will be administered, which are summarized in Table 2. Data collection will occur in person at the CBO, the participant’s house, or at another convenient location. After completing baseline data collection, participants will be told that they will be contacted as soon as enough people are enrolled to start the next cohort. Based on our prior experience, we expect wait times of under 3 weeks.

The GRT will comprise 16 cohorts with at least 9 people each. After enough eligible individuals are enrolled to populate 2 cohorts, we randomly assign them to either the immediate intervention or wait-list control cohort within that pair. For people in cohorts randomized to the immediate intervention condition, staff will provide instructions for attending the first intervention session. This will be followed by mailed written instructions and a reminder phone call 2–3 days before the first session. For people in cohorts randomized to the wait-list control condition, staff will explain the next steps, including the approximate date of the next data collection. Participants in the wait-list control arm will receive a mailed summary of the next steps, and they will periodically receive phone calls, emails, texts, or mailed letters to maintain contact for scheduling the follow-up data collection. After their study participation is completed, people in the wait-list control cohorts will have the opportunity to receive the intervention, regardless of whether they were retained for the full 12 months of data collection. Assessments take 30–45 min. Each community partner organization keeps its data records and reports the deidentified data to our shared RedCap database.

We will collect baseline and then follow-up at 3, 8, and 12 months after the date of the first hula lesson for the immediate intervention cohort. Because the first phase of our hula intervention involves only hula lessons and our previous study (Kaholokula et al., 2021) found the greatest improvements in blood pressure occurred in these first 3-months, we conducted the first follow-up at 3 months. Our decision to conduct the next follow-up at 8 months is based on a previous study suggesting that it takes at least 8 months of a lifestyle intervention to show significant cognitive improvements in persons at risk for ADRD (Rovner et al., 2018). A 3- and 8-month follow-up also allows us to capture the unique effects of each phase on overall vascular and cognitive risk factors, allowing us to observe where the most change occurs, if any. The final assessment at 12 months (our primary endpoint) will allow us to determine whether the intervention has an effect after active participation in the study has concluded.

Data collection protocols will be identical for all participants to prevent bias that could occur if data collection methods differ by study condition. To schedule the follow-up visits, participants will be contacted about 1 month before the target visit date to remind them of the upcoming data collection and inform them that staff will be making contact for scheduling. Study staff will subsequently make up to 3 attempts to contact each participant by telephone, text, or email, according to preferences specified at enrollment. If these attempts are unsuccessful, a follow-up call or letter will be mailed informing the participant that we have been trying to reach them and providing contact information to schedule the appointment. At this time, we will also reach out to alternative contacts (family or friends) using information provided by the participant at enrollment. If we have been unable to schedule the follow-up by the target date, participants will be mailed a final letter informing them that the visit will be considered as skipped. For people who miss the 3- or 6-month visit, this letter will also explain that they are still eligible for future follow-up.

All participants will receive $25 as an incentive for each data collection visit. Compensation will be provided in the form of gift cards, typically for local gas or grocery outlets, but details will be determined in consultation with stakeholders at each CBO. Participants who miss any data collection visit will have the chance to reschedule and will receive full compensation if the visit is completed within one month of the target date.

Trained research staff will conduct intermittent fidelity checks to record the degree to which *Kumu Hula* and community peer educators follow the standardized protocols, with booster training offered as needed. Attendance at hula lessons will be tracked. A Data and Safety Monitoring Plan and Board will ensure participant safety and study data validity and integrity.

#### 2.3.4. Measures

Cognitive function will be measured by the CCI, NSCT, and QDRS. Also, Cognivue,^®^ a computerized tool for the automated assessment of cognitive functioning that is not dependent on traditional question-and-answer testing, will be administered (Cahn-Hidalgo et al., 2020). SCI and MCI will be defined as described earlier using a combination of CCI, NSCT, and QDRS scores. The CCI will measure our primary outcome for subjective cognitive complaints. The CCI, NSCT, QDRS and Cognivue^®^ will measure our outcomes of change in cognitive performance. CCI, NSCT, and QDRS will be analyzed individually as continuous and categorical variables to indicate improvement, stability, or deterioration. Change in SCI or MCI will be defined similar to eligibility criteria (Table 1). For people with SCI at enrollment, worsening of condition (versus stability) will be defined as declining to MCI by demonstration of an objective decline in cognitive function but with activities of daily living still within the normal range and QDRS scores 2.0–5.5. For individuals with SCI or MCI at enrollment, the worsening of condition will also include transition to ADRD, defined by an objective decline in cognitive function, interference with daily activities, and a QDRS score ≥6. While no medical care is provided as part of this project, individuals identified as MCI or transitioning from SCI to MCI will be offered referrals to a local neurologist. A Functional Activity Questionnaire developed by one of our investigators will be administered to participants to assess activities related to independent living (e.g., managing medications) and used to evaluate early-stage disease (Mayo, 2016).

For vascular risk factors, blood cholesterol and hemoglobin A1c data will be collected from a small drop of capillary blood from a finger stick using the A1CNow^®^^+^ and CardioChek Plus Analyzer. Hemoglobin A1c reflects overall blood glucose levels over the past 2–3 months and will be measured for all participants regardless of previous diabetes diagnosis. Values will be categorized as normal (<6.0 %), diabetic in good control (6.0 %–6.9 %), diabetic in fair control (7.0 %–7.9 %), or diabetic in poor control (≥8.0 %). Participants without a pre-existing diabetes diagnosis whose hemoglobin A1c is measured between 6 % and 7.9 % will be asked to see their primary care provider as soon as possible or immediately seek medical treatment for those above 8 %. Systolic and diastolic blood pressures (mmHg) will be collected with a portable automatic blood pressure device (Omron©HEM-907XL, Omron Healthcare). Standardized protocols involve taking 3 measurements, using the last 2 measures to obtain the average systolic and diastolic blood pressure for each participant (Pickering et al., 2005). Hypertensive status will be defined as blood pressure ≥130/80 mmHg. People with a blood pressure of ≥180/120 mmHg will be referred for immediate medical treatment. Weight will be measured by an electronic scale (Seca 876) that reliably captured weight change in our prior studies. Height in cm will be collected using a stadiometer (Seca 213). Body mass index will be calculated as kg/m^2^, and obesity will be defined as a body mass index ≥30. These methods and tools for collecting clinical data have been successfully used with NHPI participants in other studies (Kaholokula et al., 2014, 2017, & 2021).

For lifestyle behaviors and psychosocial factors, the 3-item Physical Activity Questionnaire will be used to assess exercise frequency during the past month, which asks about moderate activity level, vigorous activity level, and change in activity, and used previously with NHPI (Kaholokula et al., 2014). The validated Cognitive and Leisure Activity Scale is an inventory of common activities that have been well-established as beneficial in older populations (Galvin et al., 2021). The scale assesses engagement with cognitive and physical activities with a 16-item questionnaire. A 39-item version of the Eating Habits Questionnaire will estimate the average daily fat in diet (Kristal et al., 1994), which has been used previously with NHPI (Kaholokula et al., 2014). A health behavior survey will capture the frequency and duration of tobacco and alcohol use. The Global Sleep Assessment Questionnaire includes items on bed and wake times, sleep latency, and snoring, among other factors (Roth et al., 2002). IADL will be measured with the Functional Activity Questionnaire (Pfeffer et al., 1982). Depression symptoms will be measured by the 10-item CES-D. Scores will be evaluated on a continuous scale (range 0–30), and as a binary indicator of scores ≥10.

Demographic data collected will include date of birth, biological sex, marital status, employment status, educational attainment, occupation, specific NHPI ancestry, and prior hula experience. Family history of dementia will be assessed using standard protocols that ask about having at least 1 first-degree relative diagnosed with ADRD and whether a physician had established the diagnosis of ADRD in the first-degree relative following a diagnostic evaluation (Donix et al., 2012). Bilingualism will be assessed by asking: “What is the language you were exposed to from birth?” “What language do you primarily speak at home?” and “How many languages do you speak fluently?” Neighborhood safety will be assessed with an adapted 13-item scale on walking environment, safety, and social cohesion (Mujahid et al., 2007). Social habits around recreational drinking and drug use were assessed with a modified scale from the PILI ‘Ohana Project (Kaholokula et al., 2014).

Consistent with our CBPR approach, our community partners wanted to reduce the physical and mental burden on the older NHPI adults by lessening the overall assessment duration (i.e., no longer than 1 h) and the number of assessment measures. Thus, we limited the assessments to only capturing our primary (i.e., vascular risk factors for ADRD and subjective cognitive complaints) and secondary outcomes (i.e., other measures of cognitive performance, physical function, and mental health) to answer our primary research aim, which was to determine the intervention effect on improving vascular and cognitive risk factors of ADRD. We also include measures to capture potential mediators depicted in Fig. 2, such as depression, substance use, sleep behaviors, diet, and physical activity.

#### 2.3.5. Statistical analysis and power

We will first calculate descriptive statistics and generate visual plots to assess the balance of individual-level covariates between intervention and control groups at baseline and to depict informative cross-sectional and longitudinal patterns in the data. For the intervention effects on vascular health and cognitive function, we will analyze the data using group randomized trial methods that account for clustering of individuals within intervention cohorts and appropriately modify the degrees of freedom (Murray et al., 2004). Primary outcomes are blood pressure, hemoglobin A1c, blood lipids, and body-mass-index for vascular risk factors, CCI for subjective memory complaints, QDRS for attention and memory scores for cognitive function, and the Cognivue^®^ for general cognition. CCI and QDRS will be analyzed as continuous and dichotomous variables. QDRS will be dichotomized at the recommended cut-off scores of 2–5.5 for mild and ≥ 6 moderate/severe impairment (Maruff et al., 2013). CCI will use the recommended cut-off scores for SCI as 6 ≤ and less than 6 as normal cognitive function. Secondary outcomes will be other measures of vascular risk factors, cognitive function, and other variables relevant to ADRD risk. Separate regression models will be fit for each of the outcomes with no adjustment for multiple comparisons.

Linear or logistic hierarchical models will be specified to model continuous or binary versions of the outcomes (Table 3), respectively. Normality assumptions will be assessed by Q-Q plots and transformations when appropriate. All models will include a cohort-level random intercept. Analyses will first estimate the effect of the intervention on each of the primary outcomes separately for 3, 8, and 12 months. If doing so improves model fit, we will also fit a model that includes all time points and a cohort-level random slope over time. Time (baseline and 3, 8, and 12-months) will be modeled as a categorical and continuous variable. If the model fit is not significantly different, we will retain the latter as the more parsimonious specification. All models will include a binary indicator for randomization to the immediate intervention arm. We will include a coefficient for the intervention × time interaction to estimate divergence between the 2 study arms during follow-up. This term represents the statistical test of the intervention for improving the outcome. For sensitivity analysis, we will adjust for individual-level covariates for which there is evidence of imbalance between the two study arms at baseline. Lastly, we will conduct exploratory analyses to evaluate potential mediation of the intervention effect by relevant covariates (e.g., better sleep quality as an explanation for improved cognitive function) by including the potential mediators in the model to estimate controlled direct effects of the intervention. Results for inferential analyses will be reported as mean or risk differences and ratios with 95 % confidence intervals.

For GRT, statistical power is a function of the number of groups per study arm as well as the number of participants per group (Murray et al., 2004). We estimated minimum detectable effect sizes for a GRT with 8 groups per condition, 9 participants per group and approximately 80 % retention over 12 months at an alpha = 0.05. We assumed an intra-cohort correlation of 0.01 and a within-person correlation of 0.8 or 0.5 over time. We estimated standard deviations for vascular risk factors based on data from the KāHOLO Project and on previous research for hemoglobin A1c (Sinclair et al., 2013) and cognitive outcomes (Rattanabannakit et al., 2016). Table 3 shows the estimates for 80 % power to detect mean differences in the clinical variables, which depends on correlation over time. For continuous cognitive outcome measures (Cognivue^®^, CCI, NSCT), we have 80 % power to detect a standardized effect size of 0.38–0.56 (for within-person correlation over time of 0.8 or 0.5, respectively). The NIH website estimated power using GRT power calculators (U.S. Department of Health and Human Services, n.d.).

## 3. Results

‘Ike Kupuna is a 5-year project that started in September 2021 and is expected to be completed by November 2025. The enrollment of participants began on September 2, 2022, and is planned to end on November 30, 2024. The protocols for this project have been approved by the Institutional Review Board of the University of Hawai’i (2022–00090) and are registered in ClinicalTrials.gov (NCT05534607). To date, activities to achieve Specific Aim 1 have been completed and the protocols for Specific Aim 2 presented here reflect the modifications made based on the results of Specific Aim 1. As hypothesized, the adapted hula-based intervention incorporates culturally tailored education to promote a healthy diet, culturally-based exercises to improve cognitive function, and community and social support. For Specific Aim 2, we expect the hula-based intervention to be superior to the waitlist control condition in improving vascular risk factors, subjective cognitive complaints, and objective cognitive function.

## 4. Discussion

Given the high risk of ADRD among NHPI, our ʻIKE Kūpuna project seeks to reduce this risk by designing and testing a novel, culturally grounded lifestyle intervention among NHPI with the vascular risk factors for ADHD and very early signs of cognitive problems. Our project features several important innovations. First, we test an authentic cultural practice as a paradigm-shifting health promotion and disease prevention strategy aimed at preventing cognitive decline in NHPI, a high-risk population that has been largely excluded from ADRD research. Secondly, our hula program seeks to promote healthy lifestyles consistent with NHPI values and aspirations and thus likely to lead to sustainable behavior change for at-risk NHPI individuals. Finally, the use of a culturally grounded intervention that leverages a prevalent and assessable cultural practice, such as hula, can be naturally sustainable in NHPI communities with existing resources.

A strength of this project is our use of hula, a traditional cultural practice, for ADRD prevention in NHPI. This approach has a strong theoretical foundation supported by preliminary data. Its popularity and accessibility will ensure that this adapted hula-based intervention is appealing and sustainable. The primary requirement of our programs is the presence of a trained kumu, and there are hundreds of kumu present on a global level (Mele.com, 2010). Another strength is the inclusion of both subjective and objective cognitive measurements. Considering the mild complaints many participants will report, relying solely on cognitive performance could miss important improvements in both symptoms and longer-term outcomes among individuals with SCI.

There are also some notable limitations. A potential challenge is our wait-list control design, which limits the feasible length of follow-up in the GRT. Our partner communities believe it is unethical to withhold the intervention from any participant, given its previous effectiveness for lowering blood pressure beyond 12 months. Notwithstanding, our 12-month design will permit us to assess within-person cognitive change over time, and we are mainly conducting a primary prevention study to reduce risk years before dementia onset by improving vascular risk factors for ADRD. Another potential challenge will be ensuring the consistency of the intervention across the five CBO. Thus, we will provide robust training and conduct regular fidelity checks. Additionally, we anticipate that our participant sample will include more women than men, given our previous experience with intervention research. Thus, we will strive to achieve a balanced representation of the sexes. Also, the absence of validated cognitive assessment tools in NHPI communities can result in potential measurement bias or be hindered by linguistic or cultural factors. We are currently performing factor analyses on the tools used in this study to determine its construct validity. Finally, because this research is being done in Hawai’i, where 25 % of the population are NHPI, our approach and results may not be as easily replicated or generalizable to NHPI in the continental U.S. However, as we noted earlier, there are numerous hula schools throughout the U.S.

## Figures and Tables

**Table 1:** Summary of SCI and MCI eligibility based on the three cognitive measures.

CCI	NSCT	QDRS	Eligibility
<6	≥36	<2	Not Eligible (Normal)
Any	Any	≥6	Not Eligible (mild-severe ADRD)
≥6	Any	<2	Eligible (SCI)
Any	Any	2–5.5	Eligible (MCI)

**Table 2:** Summary of data collection visits.

Measure/Variables	Time Point				
	Screening	Baseline	3 mo	8 mo	12 mo
**Cognitive Function**					
Cognitive Change Index	X			X	X
Quick Dementia Rating Scale	X			X	X
Number Symbol Coding Test	X			X	X
Cognivue		X		X	X
**Vascular Risk Factors**					
Blood cholesterol		X	X	X	X
Hemoglobin A1c		X	X	X	X
Blood pressure		X	X	X	X
Body-Mass-Index		X	X	X	X
**Lifestyle and Physical Function**					
Physical activity scale		X		X	X
Dietary		X		X	X
Social Habit		X		X	X
Sleep		X		X	X
IADL		X		X	X
**Psychosocial, Cultural, and Mental Health**					
Cognitive and leisure activities		X		X	X
Depressive symptoms		X		X	X
Depression screening	X				
**Other Variables**					
Demographics		X			
Neighborhood factors		X			X
Family History		X			X
Bilingualism		X			
Occupational Scale		X			X

**Table 3:** Power for group-randomized trial vascular outcomes.

Outcome	Minimum detectable difference between treatment arms		SD
	*ρ*_*m = 0.8*_	*ρ*_*m = 0.5*_	
Blood lipids	13.4	19.7	35
Hemoglobin A1c	0.8	1.2	2.2
Systolic blood pressure	5.8	8.4	15
Diastolic blood pressure	4.2	6.2	11
Body mass index	3.1	4.5	8

SD = standard deviation; ρ_m_ = *within-person correlation over time*.

## Abbreviations:

ADRD: Alzheimer’s disease and other related dementias
CBO: community-based organizations
GRT: group-randomized trial
NHPI: Native Hawaiians and Pacific Islanders
SCI: subjective cognitive impairment
MCI: mild cognitive impairment
